# Endomyocardial biopsy in patients with myocarditis—still justified in the CMR era? A single-centre experience

**DOI:** 10.1007/s00392-024-02574-4

**Published:** 2024-11-21

**Authors:** Katharina Seuthe, Roman Pfister, Lenhard Pennig, Ute Mons, Karin Klingel, Henrik ten Freyhaus

**Affiliations:** 1 Faculty of Medicine and University Hospital Cologne, Clinic III for Internal Medicine, University of Cologne, Cologne, Germany; 2 Institute for Diagnostic and Interventional Radiology, Faculty of Medicine and University Hospital Cologne, University of Cologne, Cologne, Germany; 3 Institute for Pathology and Neuropathology, University Hospital Tübingen, Tübingen, Germany

**Keywords:** Myocarditis, Endomyocardial biopsy, Therapeutic yield, Immunosuppressive therapy

## Abstract

**Background:**

In the past decades, cardiovascular magnetic resonance (CMR) was established as a non-invasive tool supporting the diagnosis of myocarditis and there is often reluctance in performing EMB due to potentially severe complications. We sought to identify patient subgroups that could still benefit from EMB in the CMR era.

**Methods:**

Data of patients presenting with myocarditis between 01/2016 and 06/2023 were analysed according to patient risks. Prespecified risk factors were (i) left ventricular ejection fraction (LVEF) ≤ 30%; (ii) severe arrhythmias; or (iii) pre-existing autoimmune disease. Furthermore, the subgroup of recurrent myocarditis cases was analysed separately.

**Results:**

A total of 137 patients (35.5 ± 14.8 years, 80.3% male) were included. 26/137 patients had a documented LVEF ≤ 30%, 13/137 a LVEF > 30% with at least one other risk factor and 98/137 a LVEF > 30% without risk factors. EMB was performed in 21/26 patients with LVEF ≤ 30% (80.8%), in 7/13 patients with LVEF > 30% and risk factors (53.8%) and in 16/98 (16%) patients without risk factors. EMB led to the initiation of immunosuppressive therapy in 11/28 patients with risk factors (39.3%) and in none of the patients without risk factors (0/16, 0%, *p* = 0.003). With respect to the subgroup of patients presenting with recurrent myocarditis (*n* = 10), no specific therapy was initiated.

**Conclusions:**

Due to a high therapeutic yield for initiation of immunosuppressive therapy in non-infectious myocarditis, performing EMB should be considered in all high-risk patients. In patients without clinical risk factors including cases of recurrent or relapsing myocarditis no specific therapy was initiated.

**Supplementary Information:**

The online version contains supplementary material available at 10.1007/s00392-024-02574-4.

## Introduction

Myocarditis is an inflammatory disease caused by viral infections, drug exposure (e.g. immune checkpoint inhibitors, ICI) or autoimmune disease (e.g. Systemic Lupus Erythematosus, SLE) and affects approximately 22 per 100,000 patients per year [1, 2]. Symptoms and severity of the disease can vary extremely between patients.

Approximately 75% of patients admitted with myocarditis have an uncomplicated course, whilst 2% to 9% of cases are complicated by haemodynamic instability or even present with cardiogenic shock [2]. Acute myocarditis is also a common cause of sudden cardiac death in young people [1, 3]. It is therefore of paramount importance to identify high-risk patients for implementation of monitoring or specific treatment.

Endomyocardial biopsy (EMB) is still considered the reference standard for diagnosing myocarditis [1, 2, 4]. However, cardiac magnetic resonance imaging (CMR) has emerged as a powerful non-invasive diagnostic tool for tissue characterisation in acute or chronic myocarditis [5] and a certain reluctance exists to perform EMB in clinical routine due to rare, but potentially severe complications. Nonetheless, EMB with assessment of viral infection status is crucial for initiation of specific, mostly immunosuppressive treatment.

Therefore, the role of EMB in the context of diagnosing myocarditis is still under discussion [1, 4, 6]. Based on current literature, EMB is recommended particularly in patients presenting with cardiac complications, peripheral eosinophilia, persistent or relapsing myocarditis and in the setting of ICI therapy [1, 4, 6]. However, data on the actual diagnostic yield and the impact or change of the initial therapeutic strategy based on EMB results are generally scarce. We therefore sought to study the diagnostic and therapeutic relevance of EMB in patients with myocarditis depending on clinical risk, to identify those most likely to benefit from the procedure.

## Methods

### Ethics

Due to the retrospective nature of the data, no ethical advice was required.

### Study population

Consecutive patients aged ≥ 18 years admitted to our tertiary care centre with the diagnosis of (peri-) myocarditis between January 2016 to June 2023 were included in this study.

Patients were identified through the *International Classification of Diseases*, 10th revision codes (I.40, I.41) recorded in hospital discharge forms. Only patients with histologically proven myocarditis (from EMB), with positive necrosis biomarkers (troponins or creatine kinase-MB) combined with two CMR criteria for myocarditis or with distinct clinical diagnosis (symptoms + positive biomarker after exclusion of differential diagnoses) were included. Patients with an alternative diagnosis at discharge as well as patients without elevated-troponin levels were excluded.

Clinical presentation, including differentiated symptoms, changes in electrocardiogram (ECG) and laboratory markers were documented at the time of admission.

Cardiogenic shock was defined as patients requiring inotropic therapy (e.g. dobutamine, levosimendan, milrinone) and/ or mechanical circulatory support (MCS) (Impella CP^®^, Impella^®^ 5.0, venoarterial extracardiac membrane oxygenation [VA-ECMO], or both).

Clinical risk factors were defined as follows according to current recommendations [1]: (i) LVEF ≤ 30%; and (ii) presence of severe arrhythmias (ventricular fibrillation [VF], ventricular tachycardia [VT], high-grade atrioventricular block [AV-block]), both defining severe manifestation of the disease. In addition, (iii) underlying autoimmune disease was regarded a risk factor due to the high likelihood of cardiac involvement in systemic disease. Furthermore, the subgroup of recurrent cases of myocarditis was analysed in detail. LVEF was acquired from the echocardiography report. Patients without performed echocardiography were excluded to provide consistency in the method.

### Laboratory markers

Troponin was measured as high-sensitivity troponin T (HS troponin T) at our institution, with a cut-off value of < 0.014 µg/L. The cut-off levels for C-reactive protein (CRP) and creatine kinase myocardial band (CK-MB) were < 5.0 mg/dL and < 25 U/L, respectively. Values above these thresholds were considered elevated.

For the correlation of troponin levels, the median troponin level of the cohort (0.33 µg/L) was used as a cut-off.

### Cardiovascular magnetic resonance (CMR)

CMR was performed using a whole body 1.5 T MRI system (Philips Ingenia, Philips Healthcare, Best, The Netherlands) equipped with a 28-channel coil. The standardised imaging protocol comprised 2D balanced steady-state free precession cine sequences in standard orientations (4-chamber [4Ch], 2-chamber [2Ch], 3-chamber [3Ch], and short axis [SA]) was well as T2-weighted fat-saturated black blood images in SA. T1- (using a 3(3)3(3)5 modified look-locker inversion recovery sequence) [7] and T2-mapping (employing a gradient-spin-echo sequence [8]) were acquired in SA followed by i.v. injection of Gadobutrol (Gadovist, Bayer HealthCare Pharmaceuticals, Berlin, Germany; 0.2 mmol/kg). Ten minutes after injection of gadolinium contrast, T1-mapping images were acquired in SA. T1-weighted inversion-recovery fast spoiled gradient-echo sequences were used for late gadolinium enhancement (LGE) in standard orientations (4Ch, SA, 2Ch, and 3Ch). All sequences were acquired in end-diastole during end-expiratory breath-hold. Diagnosis of myocarditis was given when according to the revised Lake Louise Criteria [9] both T1- (increased myocardial T1 relaxation time, increased extracellular volume fraction, or positive LGE) and T2- (increased myocardial T2 relaxation time or visual myocardial oedema/increased T2 signal intensity ratio) based criteria were met. Final diagnosis was based on multidisciplinary evaluation by a board-certified cardiovascular radiologist and the treating cardiology consultant. Inter-observer variability was minimised by drawing on results regular weekly interdisciplinary CMR meetings.

### Echocardiography

Transthoracic echocardiography (TTE) was performed in our echocardiography laboratory or for unstable patients in the intensive care unit with either a GE Vivid E 95 or a GE Vivid S70 (GE Vingmed, Horten, Norway). LVEF was calculated according to the modified Simpson rule in apical four‐chamber and two‐chamber views. Image analysis was performed through Tomtec Image-Arena Version 4.6, TomTec, Unterschleissheim, Germany.

### Endomyocardial biopsy (EMB)

The decision to perform EMB was based on the treating physician’s directions. For EMB sampling, a myocardial biopsy forceps (H + H. Maslanka, Tuttlingen, Germany) was used. To minimise sample error, five to six EMB samples were taken from the right-ventricular septum or the left-ventricular free wall under fluoroscopic guidance.

Choice of right- or left-sided EMB was based on the clinical characteristics of the patients. In case of MCS, EMB was performed from the right ventricle after establishment of VA-ECMO or Impella^®^. A dedicated team of experienced interventional cardiologists performed all procedures.

### Histological analysis

All histological, immunohistological, and molecular pathological analyses diagnosing myocardial inflammation and viral infections were performed at one reference centre, the Department of Cardiopathology, University Hospital Tuebingen (Tuebingen, Germany). Myocardial inflammation was defined as the detection of ≥ 14 infiltrating leukocytes/mm^2^ (CD3 + T-lymphocytes and/or CD68 + macrophages) in addition to enhanced human leukocyte antigen class II expression in professional antigen-presenting.

Acute Myocarditis requiring myocyte injury/necrosis was differentiated from healing/chronic myocarditis, which was defined by the following criteria: absence of myocyte necrosis but detection of ≥ 14 infiltrating leukocytes/mm^2^ (CD3 + T-lymphocytes and/or CD68 + macrophages), and fibrosis.

### Diagnostic and therapeutic consequence

Diagnostic consequence was defined as securing the diagnosis of myocarditis, whilst therapeutic consequence as the initiation of specific immunosuppressant therapy. The decision to start immunosuppressant therapy was based on the biopsy result after EMB and the clinical status by the treating physician considering guideline recommendations [10].

### Statistical analysis

Data analyses were performed using SPSS statistical software (version 26, IBM, Ehningen, Germany). Data are shown in absolute values, percentages, and/or means with standard deviation**s**. Proportions are expressed as number of patients and percentages. Categorical variables were compared using contingency tables and application of Fisher’s exact test. p value < 0.05 were considered as statistically significant.

## Results

### Study population

A breakdown of baseline characteristics and clinical presentation of the study population is shown in Table 1. Out of 162 patients with (peri-) myocarditis, 137 patients (35.5 ± 14.8 years, 80.3% male) met the inclusion criteria and were included in this retrospective analysis. The most frequent clinical presentations were chest pain (90/137, 65.7%), respiratory tract infection (58/137, 42.3%) and dyspnoea (51/137, 37.2%). Other, less-frequent symptoms were palpitations (10/137, 7.3%) or gastrointestinal disorder (22/137, 16.0%).

**Table 1:** Baseline characteristics and clinical presentation of patients presenting with myocarditis

	Total	%
Patients, *n*	137	
Age, mean (SD)	35.5 (14.8); range 18–82	
Female sex, *n*	27	19.7
Clinical presentation		
Chest pain	90	65.7
Dyspnoea	51	37.2
Palpitations	10	7.3
Fever	49	35.8
Respiratory tract infection	58	42.3
Gastrointestinal disorders	22	16.0
Cardiogenic shock	31	22.6
Requiring MCS	16	11.7
Electrocardiogram		
Abnormal	68	49.6
Arrhythmias	24	17.5
Atrioventricular block	4	2.9
Atrial fibrillation	7	5.1
Ventricular fibrillation	5	3.6
Ventricular tachycardia	3	2.2
ST-segment elevation	26	19.0
T-wave inversion	21	15.3
Bundle-branch block	6	4.3
Laboratory markers		
Elevated troponin	137	100
Elevated CK-MB	85	62.0
Elevated CRP	119	86.9
LVEF at admission		
≥ 50%	86	62.7
41–49%	16	11.7
31–40%	9	6.6
≤ 30%	26	19.0

SD, standard deviation; LVEF, left ventricular ejection fraction; CRP, C-reactive protein; CK-MB, creatine kinase myocardial band; MCS, mechanical circulatory support

Patients were all hospitalised with acute symptoms and met clinical criteria of acute myocarditis.

The majority of patients (86/137, 62.7%) showed a preserved left ventricular systolic function (LVEF ≥ 50%) with 26/137 (19.0%) patients showing severely impaired LV function.

Life-threatening arrhythmias occurred in 12/137 patients (8.7%) and 31/137 patients (22.6%) were admitted in cardiogenic shock, of which 16/31 patients (51.6%) required short term MCS (Impella^®^, VA-ECMO or both).

### Diagnosis of myocarditis

All patients underwent 12-lead ECG recording and blood sample testing. 68/137 patients (49.6%) presented with an abnormal ECG and findings such as ST-segment elevations (Table 1). Elevated troponin levels were found in 137/137 of patients (100%). TTE for LV- function assessment was performed in 137/137 patients (100%). In 67/137 patients (48.9%) a coronary angiogram was performed to rule out myocardial infarction as a potential alternative diagnosis. All patients in which no coronary angiogram was performed had a distinct CMR confirming the diagnosis and excluding ischaemic myocardial lesions.

In all patients (137/137, 100%), in addition to elevated-troponin levels, the diagnosis of myocarditis was based on CMR and/or EMB results.

In 110/137 patients (80.2%), a CMR was performed. A CMR was predominantly conducted in patients presenting with LVEF ≥ 50% (81/86 patients, 94.1%) whilst only 38.5% of patients (10/26) with myocarditis and an LVEF ≤ 30% underwent CMR. CMR could not be performed in these cases due to clinical instability (13/16), obesity (2/16) or previous implantation of an internal cardioverter defibrillator (1/16).

An EMB was performed in 44/137 patients (32.1%), in most cases samples were taken from the right ventricular septum (41/44). In three patients with recurrent myocarditis, CMR showed inflammation only in the left ventricle, therefore, left ventricular biopsy was performed (see Table 2). Most frequently, biopsies were taken in patients with LVEF ≤ 30% (21/26, 80.7%) followed by patients with LVEF > 30% with risk factors (7/13, 53.8%). In low-risk patients only 16/98 (16.3%) an EMB was performed, however these were still 16/44 (36.4%) of performed biopsies. Ten/16 (62.5%) of EMB in low-risk patients were performed in recurrent myocarditis cases.

**Table 2:** Detailed histological results of EMB

	Total	%	High-risk patients	Low-risk patients	p	RV-biopsy	LV-biopsy
Endomyocardial biopsies	44		21	16		41	3
Histological evidence of myocarditis	33	75				32	1
Acute myocarditis	11	25	11	0	0.003	11	0
Lymphocytic	8						
Giant cell	2						
Eosinophilic	1						
Positive virus genome	5						
EBV	2						
HHV6	2						
Parvovirus B19	1						

EBV, Ebstein Barr virus; HHV6, Human herpesvirus 6

Histological evidence of myocarditis was found in 33/44 (75%) of all patients (see Table 2). Acute myocarditis (including histological evidence of myocyte injury/necrosis) was found in 11/33 patients (33.3%), in 22/33 (66.7%) subacute or healing/chronic myocarditis was described. Histologically, lymphocytic myocarditis was the most frequent finding (28/33 patients, 84.8%). Of the acute cases, eight revealed lymphocytic myocarditis, one eosinophilic myocarditis and two giant-cell myocarditis. Eosinophilic or giant-cell myocarditis were associated with cardiogenic shock in three/three cases and ventricular tachycardia in two/three cases.

In 5/44 (11.3%) biopsies, virus DNA was detected in myocytes. Due to low copy count, no virus-specific therapy was initiated. Nevertheless, in one case virus detection prevented the initiation of immunosuppressive therapy.

Of the patients receiving EMB, 25/44 (56.8%) were also examined with CMR whilst the remaining 19/44 (43.2%) patients were unable to sustain CMR due to clinical instability and/or MCS.

### Subgroup of high-risk patients

39/137 (28.5%) patients were stratified as high-risk with at least one risk factor. Amongst these, 26/137 patients (19.0%) presented with a severely reduced LVEF (≤ 30%), whereas 13/137 (9.5%) patients with LVEF > 30% showed at least one of the predefined additional risk factors: VT (*n* = 3), VF (*n* = 3), high-grade AV-block (*n* = 2), systemic lupus erythematosus (*n* = 1) or colitis ulcerosa (*n* = 1). In 28/39 (71.7%) patients with high-risk EMB was performed.

Diagnosis of acute myocarditis by EMB/histology was established exclusively in patients with LVEF ≤ 30% (8/21, 38.1%), and in patients with LVEF > 30% with risk factors (3/7, 42.9%), versus 0/16 in the low-risk cohort (*p* = 0.003).

**Fig. 1:**
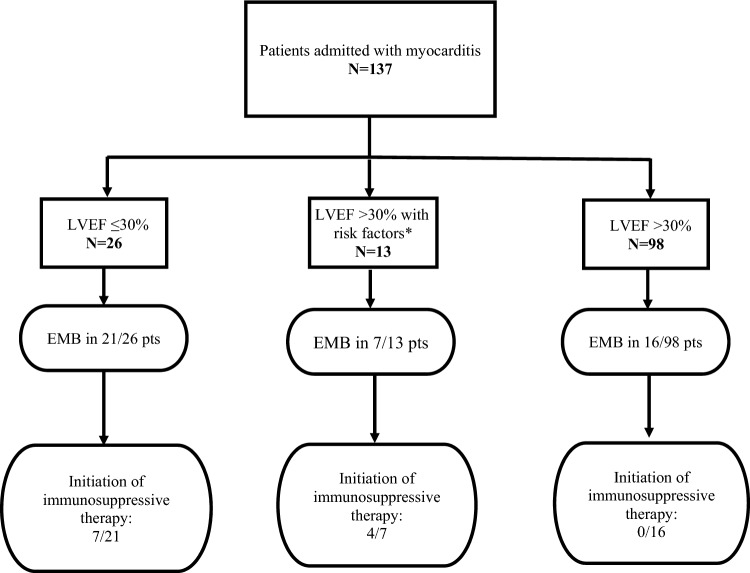
Frequency and therapeutic yield (initiation of immunosuppressive therapy) of EMB depending on patient risk factors. EMB, endomyocardial biopsy; LVEF, left ventricular ejection fraction; VF, ventricular fibrillation; VT, ventricular tachycardia; AV-block, atrioventricular block; pts, patients. *Defined risk factors: presence of severe arrhythmias (VF, VT, high-grade AV-block) or underlying autoimmune disease

Figure 1 depicts the distribution of patients in high- and low-risk as well as the results of EMB in these groups.

### Therapeutic consequences of EMB

EMB had direct therapeutic consequence leading to initiation of specific immunosuppressive therapy in 11/44 patients (25%) who were all part of the high-risk groups with LVEF ≤ 30% (*n* = 7) or with LVEF > 30% and risk factors (*n* = 4). Consequently, the therapeutic yield for EMB leading to immunosuppressant therapy was 39.3% in high-risk patients (11/28). In contrast, EMB had no therapeutic consequence in any of the low-risk patients (*p* = 0.003). Immunosuppressant therapy was initiated with prednisolone monotherapy (7/11) in cases of acute or subacute lymphocytic and one case of eosinophilic myocarditis. Prednisolone in combination with azathioprine (2/11) was administered in cases of chronic lymphocytic myocarditis with LVEF < 30% and prednisolone in combination with cyclosporine and mycophenolatmofetil (2/11) in the cases of giant-cell myocarditis according to current recommendations [1]. Figure 1 depicts the distribution of therapeutic consequence.

Regarding troponin as a potential risk factor, there was no significant difference in patients with high troponin and low troponin regarding the diagnosis of acute myocarditis or therapeutic consequence of EMB (Table 4, supplement).

### Subgroup of patients with recurrent myocarditis

In 13/137 patients, a recurrent myocarditis was diagnosed and in 10/13 patients EMB was performed. Consequently, 10/44 patients that were subjected to EMB had a recurrent myocarditis, of those all had a normal or mildly impaired LVEF (≥ 50% or 41–49%). Histology showed either healing or chronic lymphocytic myocarditis or no evidence of inflammation. Based on these results, the clinically stability of these patients as well as the preserved LVEF in most cases, the clinical decision was made not to initiate specific therapy. Detailed information on the subgroup of recurrent myocarditis is depicted in Table 3.

**Table 3:** Detailed information on patients with recurrent myocarditis

	Total	%
Patients with recurrent myocarditis, *n*	13	9.5%
Age, mean (SD)	33.1 (10.3)	
Female sex, *n*	1	7.7
Clinical presentation		
Chest pain, *n*	9	69.2
Dyspnoea, *n*	3	23.0
Palpitations, *n*	2	15.3
Syncope, *n*	1	7.7
Arrhythmias, *n*	0	0
Cardiogenic shock, *n*	0	0
LVEF at admission		
≥ 50%	9	69.2
41–49%	4	30.8
31–40%	0	0
≤ 30%	0	0
Diagnostics		
TTE, *n*	13	100
CMR, *n*	13	100
EMB, *n*	10	76.9

SD, standard deviation; TTE, transthoracic echocardiography; CMR, Cardiovascular magnetic resonance, EMB, endomyocardial biopsy

### Complications of EMB

In 2/44 (4.5%) patients, a pericardial tamponade occurred after EMB which resolved without sequelae after drainage. No complications occurred in unstable patients or patients on MCS requiring EMB.

### Follow-up and mortality

The in-hospital mortality was 4.4% in the whole study population, 13.6% in patients that underwent EMB and 0% in patients without EMB, respectively. All patients who died in the hospital were part of the high-risk group (LVEF ≤ 30%, or LVEF > 30% plus other risk factors [VT, VF, high-grade AV-Block, systemic autoimmune disease]), and histological examination of their EMB samples revealed either acute lymphocytic myocarditis, eosinophilic myocarditis, or giant-cell myocarditis. Consequently, the in-hospital mortality was 15.4% in the group of high-risk and 0% in the group of low-risk patients, respectively. Follow-up data were available for 121/137 patients. During follow-up, one more patient in the high-risk group deceased within one year resulting in a 1-year mortality of 5.1% in the whole study population, 17.9% in the high-risk cohort and 0% in low-risk group, respectively.

## Discussion

### Main findings

Our study provides several important findings:

1. In hemodynamically compromised patients or patients who are unable to undergo CMR due to severely compromised clinical status, EMB is safe and effective to confirm/establish the diagnosis.
2. EMB results had therapeutic consequence exclusively in high-risk patients (LVEF ≤ 30%, or LVEF > 30% plus other risk factors [VT, VF, high-grade AV-Block, systemic autoimmune disease]). Furthermore, only in this group of patients, acute myocarditis was diagnosed by histology.
3. On the contrary, in (i) low-risk patients with LVEF > 30% without additional clinical risk factors, even in (ii) recurrent or relapsing myocarditis the clinical decision was made against immunosuppressive therapy and EMB did not alter patient management.

### Generalizability of the study population

The study population represents a typical myocarditis cohort with the baseline characteristics being in line with published literature [1, 2, 11, 12]. Interestingly, as compared to other registries [2, 13, 14], the number of patients presenting with cardiogenic shock (31/137, 22.6%) including 16/137 patients (11.7%) requiring MCS (VA-ECMO, Impella® or both) was higher in our study. This discrepancy is explained by the role of our institution as a tertiary care and VA-ECMO centre.

Whilst the majority of our patients (62.7%) presented with LVEF ≥ 50%, Younis et al. [15] described even a higher number of patients (260 of 322, 81%) with a preserved ejection fraction, which again suggests that our patient cohort represents a more severely compromised collective as compared to the published literature. Similarly, the in-hospital mortality rate in our cohort was slightly higher (4.4%) compared to the 2.3% reported by Ammirati et al. [13].

Mortality was notably higher in patients with risk factors (15.4%) compared to those without any risk factors (0%). These findings are consistent with the literature.

### The diagnostic role of EMB in myocarditis patients

Whilst significantly less patients underwent EMB (44/137) than CMR (110/137) for confirmation of the diagnosis of myocarditis, the absolute frequency of EMB was high in the light of published literature. For example, whilst 31.1% of patients underwent EMB in our cohort, in a study by Ammerati et al. [13], only 12% of patients with acute myocarditis underwent EMB. In the subgroup of patients with LVEF ≤ 30%, histological sampling and analysis were performed at even higher numbers, as 80.7% of patients underwent EMB. In contrast, CMR was only performed in 38.5% of patients within this group. These data highlight the diagnostic importance of EMB in this subset of patients, which of course cannot be generalised to low-risk of the general myocarditis population.

However, still 36.4% of EMB were performed in patients with low risk, mostly in recurrent cases and cases referred for second opinion.

### Safety and efficacy of EMB

Histological evidence of myocarditis was found in 75% of cases. In two cases, a procedure-related pericardial effusion occurred (4.5%), both resolved after percutaneous drainage without sequelae. In the context of myocarditis, this complication rate is similarly compared to the literature [16]. Interestingly, the complication rate of EMB in this subset of patients appears to be higher as compared to other clinical settings, such as cardiomyopathies [17].

The majority of EMBs at our tertiary centre were performed from the RV, particularly as this is the standard approach for monitoring allograft rejection. However, there is variability in the choice of biopsy site (LV vs. RV) for myocarditis in the literature, which often depends on the centre’s protocols and the operator’s expertise. The mode of complications varies based on the biopsy site: RV biopsies carry a higher risk of cardiac tamponade, whilst LV biopsies are associated with a greater risk of systemic embolic events [18]. However, there is no definitive consensus on a standard recommendation favouring one site over the other in the context of myocarditis. As some studies suggest that LV biopsies may offer a higher diagnostic yield [17, 18], an increased application of LV biopsy in myocarditis patients can be expected in the future.

In contrast to previous publications [19], none of these complications occurred in patients on short-term MCS, especially VA-ECMO, despite the higher risk of bleeding in the context of anticoagulation therapy during MCS and potential risks during patient transfer to the catheterization laboratory, confirming the safety of EMB even in the most compromised subset of patients within an experienced team.

### The role of EMB in low-risk patients

In low-risk patients with LVEF > 30% and no additional risk factor, the predominant diagnostic tool to confirm the clinical diagnosis was CMR. This is in line with reported data describing a higher diagnostic yield of EMB in patients with severe presentation of symptoms [20]. We confirm previously published data from low-risk cohorts [13], as in none of these patients EMB revealed an acute myocarditis or had any therapeutic consequence in our low-risk patients.

### The role of EMB in high-risk patients

The highest yield (39.3%) in terms of therapeutic and diagnostic consequence was seen in patients with LVEF ≤ 30%, and in patients with LVEF > 30% and presence of additional risk factors such as high-grade AV-Block, ventricular arrhythmia or underlying autoimmune disease. The observed yield of therapy initiations in our study was similar to previously described data in the high-risk cohorts in the context of new onset of heart failure [20] and myocarditis [13] where other risk factors were defined.

Our findings therefore add objective parameters for the identification of patients with high risk of acute (fulminant) myocarditis. Whilst LVEF < 50%, ventricular arrhythmias and fulminant presentation and sudden onset of symptoms were previously suggested [20], our data allow us to further specify these criteria. We propose including LVEF ≤ 30% and add high-grade AV-block as part of the criteria to identify a subgroup of patients that has the highest diagnostic and therapeutic benefit from EMB.

One factor that might influence these observations is the extent of the inflammatory infiltrate: the more extensive the infiltration, the higher the likelihood of a diagnostic finding in EMB. Fulminant myocarditis causing severe depression of LV function is typically associated with diffuse inflammatory infiltration and is more easily identified in histology. In contrast, patients with preserved LVEF, are likely to have less diffuse and more focal infiltrates, which may be prone to sampling error or more difficult to detect histologically [4].

Another factor influencing the diagnostic performance of histology is the time from the onset of symptoms until performance of EMB. This notion is supported by the finding that in healing/chronic myocarditis, infiltrates are sparse and a larger amount of fibrosis is present, thereby increasing the probability of a non-diagnostic EMB [4]. To overcome these shortcomings and to enhance diagnostic accuracy, new promising methods like electroanatomic mapping are evolving [21].

Our histopathological findings were coherent with other high-risk cohorts, lymphocytic myocarditis being the most frequent diagnosis [22]. Severe conditions like eosinophilic or giant-cell myocarditis were all found in patients presenting in cardiogenic shock and/or on short term MCS as well as ventricular tachycardia (2 out of 3). This is in line with published reports stating that ventricular arrhythmias are often found in patients with giant-cell myocarditis with a prevalence of 29% and 55%, respectively [23, 24].

Our data suggest limited diagnostic benefit of EMB for patients who were clinically stable enough to have a CMR performed. Especially in ICI myocarditis, CMR may be inconclusive, as published data suggest caution in reliance on LGE or a qualitative T2-STIR-only approach for the exclusion of ICI-associated myocarditis [25]. Therefore, EMB should be considered in CMR-negative patients with clinically suspected myocarditis, when results may indeed change patient management. On the other hand, patients with a clear diagnosis on CMR but with high-risk features, still may benefit from EMB as only assessment of the histological subtype enables establishment of a targeted therapeutic approach.

### The role of EMB in recurrent myocarditis

Interestingly, in all patients with recurrent myocarditis, based on clinical status and the EMB results, the treating physician voted against the initiation of immunosuppressive therapy. Of note, no patient in this group presented with arrhythmias, underlying immune disease, or other above-mentioned risk factors. To the best of our knowledge, there is no existing data on this subgroup, but given the clear results of our study, despite the small sample size, it seems to be reasonable to assume that EMB in recurrent myocarditis could in the future be restricted to high-risk cases. The clinical decision regarding the initiation of specific therapy might have differed in other centres.

## Limitations

The present study is a single-centre, retrospective study with all its inherent limitations, most importantly the proneness to bias with respect to patient selection. We tried to address this issue by specifying clear inclusion and exclusion criteria as specified in the methods section. Furthermore, the high amount of patients receiving EMB and the number of patients with cardiogenic shock may not necessarily reflect patient populations in first and secondary care centres. In addition, the results on patients with recurrent myocarditis are limited given the low number of respective cases in this study. Hence, further studies, preferably in a multi-centre setting are required to perform the findings of this work.

## Conclusions

EMB remains an important tool in suspected acute myocarditis, particularly in unstable patients unable to undergo CMR. Given its high therapeutic yield for immunosuppressant therapy, EMB should be considered in all myocarditis patients with LVEF ≤ 30% or LVEF > 30% with additional risk factors (ventricular tachycardia, high-grade AV-Block or systemic autoimmune disease). In low-risk patients with recurrent/relapsing myocarditis EMB did not change patient management at our centre.

## Supplementary Information

Below is the link to the electronic supplementary material.

Supplementary file1 (DOCX 13 KB)

## Data Availability

All data are available upon contacting the corresponding author.
