# Supplementary material for: Endomyocardial biopsy in patients with myocarditis—still justified in the CMR era? A single-centre experience
Source: Clin Res Cardiol. 2024 Nov 21;115(5):743–51. doi: 10.1007/s00392-024-02574-4 (PMC13083329; doi:10.1007/s00392-024-02574-4)
Supplement: Supplementary file 1 — Supplementary file1 (DOCX 13 KB) [file 392_2024_2574_MOESM1_ESM.docx]

**Supplementary Material**

|  | Patients with low troponin levels, n= 27 | Patients with high troponin levels, n= 17 | p- value |
| --- | --- | --- | --- |
| Histology of acute myocarditis in EMB | 4 (14.8 %) | 7 (41.2%) | 0.075 |
| Therapeutic consequence of EMB | 4(14.8% | 7 (41.2%) | 0.075 |
| **Table 4:** Analysis of high vs low troponin levels (cut off = median of the cohort of 0.33 µg/L) of the patients undergoing EMB. P < 0.05 considered significant.EMB= endomyocardial biopsy | | | |
